# Photodegradation assessment of ciprofloxacin, moxifloxacin, norfloxacin and ofloxacin in the presence of excipients from tablets by UPLC-MS/MS and DSC

**DOI:** 10.1186/1752-153X-7-133

**Published:** 2013-07-31

**Authors:** Urszula Hubicka, Paweł Żmudzki, Przemysław Talik, Barbara Żuromska-Witek, Jan Krzek

**Affiliations:** 1 Department of Inorganic and Analytical Chemistry, Jagiellonian University Medical College, Faculty of Pharmacy, 9 Medyczna Street, 30-688 Kraków, Poland; 2 Department of Medicinal Chemistry, Jagiellonian University Medical College, Faculty of Pharmacy, 9 Medyczna Street, 30-688 Kraków, Poland

**Keywords:** Ciprofloxacin, Moxifloxacin, Norfloxacin, Ofloxacin, Photodegradation, Kinetic evaluation, Ultra performance liquid chromatography, Tandem mass spectrometry, Differential scanning calorimetry

## Abstract

**Background:**

Ciprofloxacin (CIP), moxifloxacin (MOX), norfloxacin (NOR) and ofloxacin (OFL), are the antibacterial synthetic drugs, belonging to the fluoroquinolones group. Fluoroquinolones are compounds susceptible to photodegradation process, which may lead to reduction of their antibacterial activity and to induce phototoxicity as a side effect. This paper describes a simple, sensitive UPLC-MS/MS method for the determination of CIP, MOX, NOR and OFL in the presence of photodegradation products.

**Results:**

Chromatographic separations were carried out using the Acquity UPLC BEH C_18_ column; (2.1 × 100 mm, 1.7 μm particle size). The column was maintained at 40°C, and the following gradient was used: 0 min, 95% of eluent A and 5% of eluent B; 10 min, 0% of eluent A and 100% of eluent B, at a flow rate of 0.3 mL min^-1^. Eluent A: 0.1% (v/v) formic acid in water; eluent B: 0.1% (v/v) formic acid in acetonitrile. The method was validated and all the validation parameters were in the ranges acceptable by the guidelines for analytical method validation. The photodegradation of examined fluoroquinolones in solid phase in the presence of excipients followed kinetic of the first order reaction and depended upon the type of analyzed drugs and coexisting substances. Photodegradation process of analyzed drugs was confirmed by differential scanning calorimetry. In addition, the identification of degradation products was carried out by mass spectrometry.

**Conclusion:**

The developed UPLC-MS/MS method enables the determination of CIP, MOX, NOR and OFL in the presence of photodegradation products and identification of photodegradation products.

## Background

Ciprofloxacin (CIP), moxifloxacin (MOX), norfloxacin (NOR) and ofloxacin (OFL), are the antibacterial synthetic drugs, belonging to fluoroquinolones group. Fluoroquinolones exhibit increased antibacterial activity against the Enterobacteriaceae and other Gram-negative bacteria such as Pseudomonas aeruginosa, and exert some activity against certain Gram-positive cocci [1,2]. Among them, MOX also show notable efficacy against atypical bacteria of Chlamydia spp., Mycoplasma spp., and Legionella spp. genera, bacilli of genus Mycobacterium and anaerobe of Bacteroides and Clostridium genera [3-5].

Fluoroquinolones are compounds susceptible to photodegradation process, which may lead to reduction of their antibacterial activity and to induce phototoxicity as a side effect [6]. In the available literature many papers devoted to the studies of fluoroquinolone photodegradation in solutions can be found [7-10], while only a few relate to photodegradation studies of a substance or of the pharmaceutical preparation in the solid phase [10-13].

The photodegradation process of CIP in solid phase was analyzed by thin layer chromatography method with densitometric detection in the presence and without selected metal ions. Samples were prepared on Petri dishes by evaporation of the test solution to obtain a dry residue. They were exposed to UV radiation within the range from 240 nm to 280 nm for 96 h [11]. Assessed the studies performed it was found that both ion concentration and ion type have an effect on the degradation process that leads to the generation of two photoproducts. The chemical structures of the photodegradation products were identified as follows 7-[(2-aminoethyl)amino]1-cyclopropyl-6-fluoro-1,4-dihydro-4-oxo-qunoline-3-carboxylic acid and 7-amino-1-cyclopropyl-6-fluoro-1,4-dihydro-4-oxo-qunoline-3-carboxylic acid [11].

Photostability of MOX after UVA irradiation in solutions and solid phase, with and without participation of Cu(II), Zn(II), Al(III), and Fe(III) was tested. Samples for testing in solid phase were prepared in the same manner as described in a publication on CIP [11]. They were exposed to UVA radiation within the range from 320 nm to 400 nm for 15 h [10]. The studies were carried out by the TLC-densitometric method. In solid phase, all metal ions decreased the photodegradation of MOX. Identification of the degradation three products was performed with LC-MS/MS and ^1^HNMR identified them as: 1-cyclopropyl-6-fluoro-7-amino-8-methoxy-4-oxo-1,4-dihydroquinoline-3-carboxylic acid, 1-cyclopropyl-6-fluoro-8-methoxy-4-oxo-7-(2-oxo-octahydro-6H-pyrrolo[3,4-b]pyridine-6-yl)-1,4-dihydroquinoline-3-carboxylic acid, 7-[3-hydroxyamino-4-(2-carboxyethyl)pyrrolidin-1-yl]-1- cyclopropyl-6-fluoro-8-methoxy-4-oxo-1,4-dihydroquinoline-3-carboxylic acid [10].

Photodegradation of NOR in solid phase in the presence of different excipients used for making tablets has been studied. The NOR tablets were exposed under direct sunlight, fluorescent light and UV radiation (continuous 254-nm UV lamp), at ambient temperature (25°C) and 65% relative humidity. The separation of NOR and its degradation products was achieved isocratically using a Lichrosorb® C8 chromatographic column (10 μm, 20 cm ×4.6 mm) and phosphate buffer solution adjusted to pH 3.0 – acetonitrile (85:15, v/v) as the eluent, pumped at a flow rate of 2.0 ml/min. The authors proved that during photodegradation diamineethyl derivative of NOR occurs [12].

The photostability of OFL in the solid state has been investigated. Irradiation was conducted in a chamber SUNTEST CPS + at 30°C using xenon lamp (1.5 kW) equipped with a 6 mm special glass filter, transmitting light corresponding to exposure behind window-glass (cut-off approximately 310 nm). The change in colour of uncoated and film coated OFL tablets and compressed OFL was studied as a function of irradiance level and total exposure energy. The degradation of OFL was quantified by HPLC. The separation of OFL and its two degradation products was achieved using a Symmetry C-18 chromatographic column (3,5 μm, 100 mm × 4.6 mm). The mobile phase consisted of triethylamine (4.5 ml), double-distilled water (830 ml) and acetonitrile (140 ml). The pH was adjusted to 2.3 with 85% H_3_PO_4_ before addition of acetonitrile. The flow rate was 1 ml/min. The structure of two main degradation products of OFL has been postulated from LC-MS analysis [13].

In summary, the existing research concerning the photostability of fluoroquinolones in the solid phase are not a comprehensive but relate of individual substances. The study was performed using different irradiation conditions and exposure time. Only two papers concern the influence of excipients on the photodegradation process. Applied methods such as TLC, HPLC and LC-MS allowed the separation and identification of up to three photodegradation products. In any paper not carried out studies of fluoroquinolones irradiated in the solid phase by DSC.

In the presented paper we have developed and validated an ultra-performance liquid chromatography method coupled with tandem mass spectrometry (UPLC-MS/MS) for the determination of CIP, MOX, NOR and OFL, which was used to study the effect of UVA irradiation on the photostability of studied fluoroquinolones in powdered tablets. In addition, the kinetic evaluation of the photodegradation process was carried out and photodegradation products have been identified. In the presented studies next to UPLC-MS/MS method, differential scanning calorimetry (DSC) was also used to compare changes in analyzed samples before and after irradiation.

## Experimental

### Chemicals and reagents

Ciprofloxacin hydrochloride monohydrate Cat. No. 91033-1G Fluka, Moxifloxacin hydrochloride series Strasbourg Cedex; Council of Europe – EDQM CS; Cat No. T30026 F-6081, Norfloxacin Cat. No. 9890-1G Fluka, Ofloxacin Cat. No. O8757-1G Sigma. HPLC grade methanol, acetonitrile and formic acid (98%) were purchased from J.T. Baker. HPLC grade water was obtained from HLP 5 (HYDROLAB Poland) apparatus and was filtered through 0.2 μm filter before use.

### Standard solution

For method validation, solutions containing different concentrations of the examined fluoroquinolones in the range 0.04 – 2.00 mg mL^-1^ were prepared.

### Pharmaceutical preparations

Proxacin 500 – film-coated tablet containing 500 mg of CIP (Polfa S.A, Poland). Excipients: pregelatinized starch, microcrystalline cellulose, colloidal silica hydrated, magnesium stearate, croscarmellose sodium, hypromellose, macrogol 6000, titanium dioxide.

Norflox- AZU – film-coated tablet containing 400 mg of NOR (Azupharma, Germany). Excipients: microcrystalline cellulose, hypromellose, magnesium stearate, poly(O-carboxymethyl) starch sodium salt, povidone, propylene glycol, colloidal silica anhydrous, talc, titanium dioxide.

Avelox - film-coated tablet containing 400 mg of MOX (Bayer, Germany). Excipients: microcrystalline cellulose, croscarmellose sodium, lactose monohydrate, magnesium stearate, hypromellose, macrogol 4000, ferric oxide, titanium dioxide.

Zanocin - film-coated tablet containing 200 mg of OFL (Ranbaxy, Indie). Excipients: microcrystalline cellulose, maize starch, lactose, magnesium stearate, polysorbate 80, talc, colloidal silica anhydrous, sodium starch glycolate, hydroxypropylmethylcellulose, macrogol 4000, titanium dioxide.

### Irradiation conditions and preparation of samples

Irradiation was conducted in a climatic chamber KBF-ICH 240 APT.line™; (Binder GmbH, Tuttlingen, Germany) at 20°C and 60% relative humidity using UVA radiation (320–400 nm) with maximum emission at 365 nm. The distance of the samples to radiation source was 13 cm. The UVA dose was determined by means of radiometer type VLX-3 W, Vilber Lourmat, with a sensor CX-365, to be each time of 5.09 × 10^-3^ J cm^-2^ min^-1^.

Powdered tablet in the amount of 50.0 mg was distributed evenly on a quartz Petri dish with a diameter of 5 cm, covered with a quartz cover and placed in the climatic chamber. The samples were exposed to UVA radiation from 7 to 113 days.

For each sample a dark control sample was prepared, which was protected with aluminum foil before irradiation.

After exposure specified number of days the tablet powder was extracted with methanol and diluted to obtain a solution having a concentration of 1.00 mg mL^-1^.

### UPLC/MS/MS analysis

The UPLC-MS/MS system consisted of a Waters ACQUITY® UPLC® (Waters Corporation, Milford, MA, USA) coupled to a Waters TQD mass spectrometer (electrospray ionization mode ESI-tandem quadrupole). Chromatographic separations were carried out using the Acquity UPLC BEH (bridged ethyl hybrid) C_18_ column; 2.1 × 100 mm, and 1.7 μm particle size. The column was maintained at 40°C. The following gradient was used: 0 min, 95% of eluent A and 5% of eluent B; 10 min, 0% of eluent A and 100% of eluent B, at a flow rate of 0.3 mL min^-1^. Eluent A: 0.1%(v/v) formic acid in water; eluent B: 0,1% (v/v) formic acid in acetonitrile. Chromatograms were recorded using Waters eλ PDA detector. Compound concentration (%i) after photodegradation was calculated from quotient of peak area (Ai) to the sum of all peak areas (∑A) on chromatograms according to formulation %i = (Ai/∑A)100 at λ = 294 nm. Spectra were analyzed in 200–700 nm range with 1.2 nm resolution and sampling rate 20 points/s. MS detection settings of Waters TQD mass spectrometer were as follows: source temperature 150°C, desolvation temperature 350°C, desolvation gas flow rate 600 L h^-1^, cone gas flow 100 L h^-1^, capillary potential 3.00 kV, cone potential 20 V. Nitrogen was used for both nebulizing and drying gas. The data were obtained in a scan mode ranging from 50 to 1000 m/z in time 0.5 s intervals; 8 scans were summed up to get the final spectrum. Collision activated dissociations (CAD) analyses were carried out with the energy of 30 eV, and all the fragmentations were observed in the source. Consequently, the ion spectra were obtained by scanning from 50 to 600 m/z range. Data acquisition software was MassLynx V 4.1 (Waters).

### DSC analysis

The DSC measurement were performed in an atmosphere of nitrogen with a flow rate of 50 mL min^-1^ using EXTAR DSC 7020 apparatus (SII NanoTechnology Inc.) equipped with DSC 7020 electric cooling unit. The temperature calibration was done with indium and tin (melting temperature T_m_ of In was 156.6°C, T_m_ of Sn was 231.88°C). The calibration of enthalpy change was done with tin (melting enthalpy ΔH_m_ of Sn was 60.46 J g^-1^).

The samples of about 4.0 to 4.9 mg were correctly weighed in an aluminum pans and sealed. Then the pans were equilibrated at 30°C for 15 min and thereafter the melting behavior were analyzed at heating rate of 10°C.

### Validation of UPLC/MS/MS method

The described method was validated for the determination of CIP, MOX, NOR and OFL in the presence of photodegradation products by UPLC method according to ICH guidelines [14]. To demonstrate the specificity of the developed UPLC method the solutions of CIP, MOX, NOR, OFL after photodegradation were analyzed. Peak purity test was carried out for CIP, MOX, NOR, OFL peaks by using MS detector in stressed sample. The system suitability parameters were defined with respect to resolution of examined fluoroquinolones peaks using solutions of CIP, MOX, NOR, OFL, after photodegradation. The linearity for CIP, MOX, NOR, OFL was assessed by injecting six separately prepared solutions covering the range of 0.18 – 2.00 mg mL^-1^. The slope of regression line, y-intercept, standard deviation of slope and intercept, correlation coefficient, R^2^ value and standard error of residuals of the calibration curve were calculated using the program Statistica v. 10. Next, to determine whether the residuals have normal distribution, the Shapiro-Wilk statistical test was used. Based on the standard error of residuals (Se) and the slope (a) of the calibration plots and following the formula LOD = 3.3Se/a and LOQ = 10Se/a, the LOD and LOQ for examined fluoroquinolones were estimated. The repeatability of the method was checked by a sixfold analysis of the concentration level 1.00 mg mL^-1^ of CIP, MOXI, NOR, OFL solutions. The same protocol was followed for three different days to study the intermediate precision of the proposed method. Different analysts prepared different solutions on different days. The RSD (%) of the peak area of examined fluoroquinolones was calculated. To demonstrate the robustness of the method deliberate small changes of flow rate, content of acetonitrile and column temperature were made around the optimal values. The mobile phase flow rate was 0.30 mL min^-1^; to study the effect of the flow rate on resolution, the flow rate was changed to 0.27 and 0.33 mL min^-1^. The effect of the column temperature was studied at 36°C and 44°C (instead of 40°C).

## Results and discussion

Photochemical reactivity of pharmaceutical substances is important in drug formulation technology. Undesirable changes in the pharmaceutical products induced by UV–VIS irradiation may occur not only during the production of active pharmaceutical ingredient (API), but also during the preparation of dosage form or during storage [15-17]. Literature data indicate that the stability of pharmaceutical substances also depends on the application of appropriate excipients [12]. Drug forms are designed by manufacturers to maintain the quality of pharmaceuticals under the storage conditions. However, the tablets are sometimes pulverized to powders in clinical use to make them easy to ingest for elderly or disabled persons if they cannot be consumed in their whole form [18].

The current guidelines of the International Conference on Harmonization (ICH) require the development of stability-indicating assay methods suitable for the determination of drugs [14].

### Method validation

Herein we developed a universal UPLC-MS/MS stability-indicating method for the separation, identification and determination of examined fluoroquinolones in the presence of its photodegradation products. The method was used for the kinetic studies of CIP, MOX, NOR and OFL.

**Figure 1:**
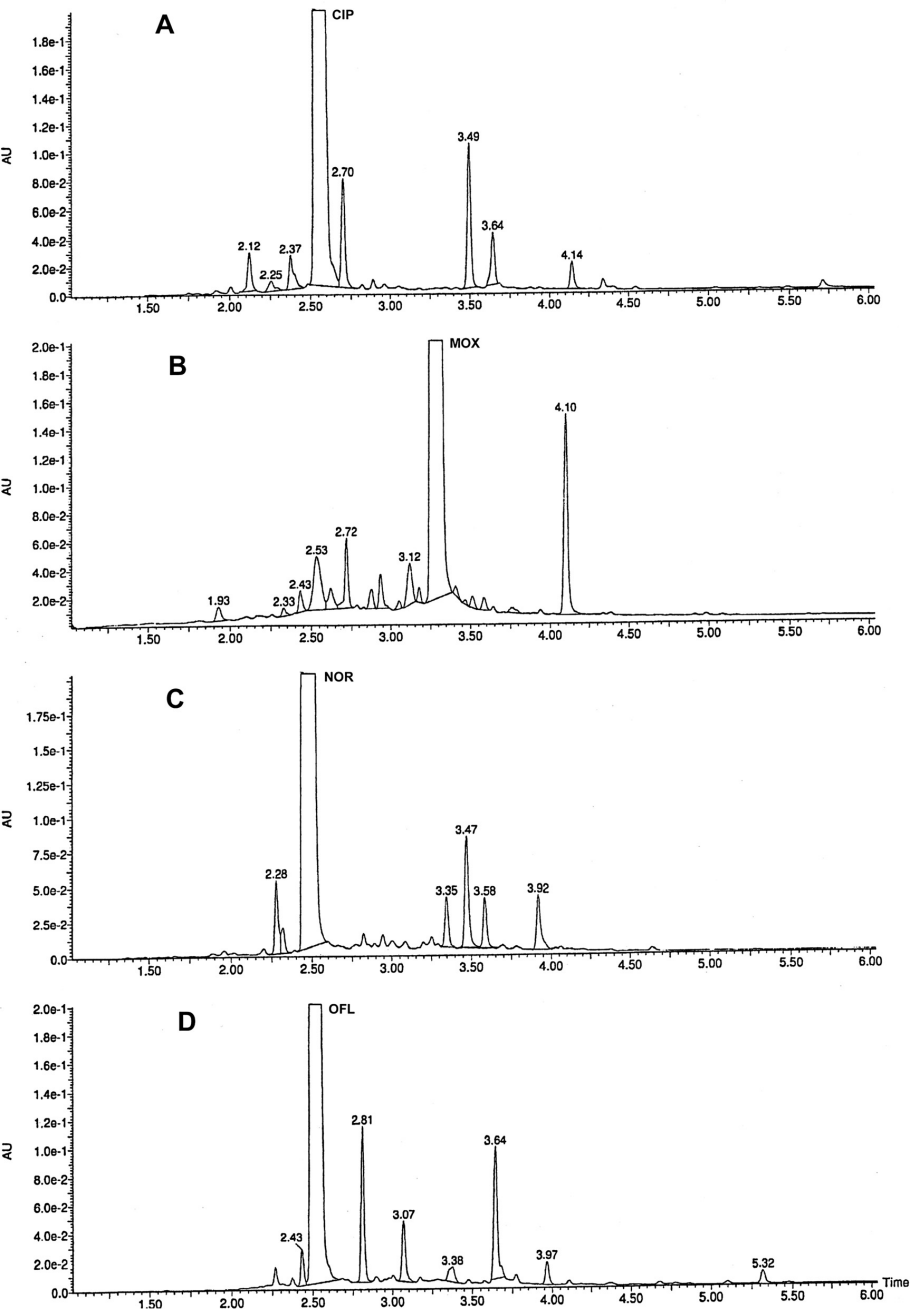
UPLC chromatograms registered for fluoroquinolones after photodegradation in solid phase in the presence of excipients: A – CIP RT = 2.53, B – MOX RT = 3.26, C – NOR RT = 2.45, D – OFL RT = 2.50.

The developed UPLC method was specific to examine fluoroquinolones and guaranteed obtaining well shaped peaks both for active substances and coexisting photodegradation products. Peaks of main components were quite well resolved from photodegradation products in chromatograms and no interference that could have an influence on the obtained results was possible (Table 1). The main peak purity was examined with MS spectra using CODA algorithm (Waters Corporation, Milford, MA, USA). The investigated MS spectra uniquely contained signals corresponding to the examined fluoroquinolones and solvent. Satisfactory resolution was also obtained for photodegradation products, peaks appearing in chromatograms were sufficiently well resolved and could be analyzed by mass spectrometry (Figure 1).

**Table 1:** Validation of the method

**Parameter**	**CIP**	**MOX**	**NOR**	**OFL**
**RT (min)**^**a**^	2.52 ± 0.05	3.22 ± 0.05	2.44 ± 0.05	2.47 ± 0.04
**Resolution**^**a,b**^	1.50	0.92	1.51	0.80
**LOD (mg mL**^**-1**^**)**	0.06	0.06	0.09	0.10
**LOQ (mg mL**^**-1**^**)**	0.18	0.18	0.26	0.30
**Linear range (mg mL**^**-1**^**)**	0.18 – 2.00	0.18 – 1.50	0.26 – 2.00	0.30 – 1.50
**Regression equation (y):**				
**Slope (a ± S**_**a**_**)**	139288.9 ± 1140.1	286408.6 ± 3181.1	166591.5 ± 2043.2	284748.3 ± 5341.4
**Intercept (b ± S**_**b**_**)**	−635.2 ± 1176.9	4474.6 ± 2691.5	−228.6 ± 2067.2	4043.2 ± 4455.5
**t = b/S**_**b**_	0.54 < t_α,f_ statistically insignificant	1.66 < t_α,f_ statistically insignificant	0.11 < t_α,f_ statistically insignificant	0.91 < t_α,f_ statistically insignificant
**Normality of residuals**^**c **^**(Shapiro-Wilk test)**	0.9343 (p = 0.49)	0.9619 (p = 0.81)	0.9387 (p = 0.54)	0.9462 (p = 0.62)
**Correlation coefficient**	0.9997	0.9995	0.9994	0.9986
**R**^**2 **^**value**	0.9994	0.9989	0.9986	0.9968
**Precision (% RSD)**	1.04	0.74	1.09	0.64
**Intermediate precision (% RSD)**	1.30	0.99	1.18	0.80

Regression equation y = ac + b; c - concentration of solution; y- peak area; S_a_ – standard deviation of slope;S_b_ - standard deviation of intercept, t- calculated value of Student’s t-test, t_α,f_ =2.776 critical value of Student’s t-test for degrees of freedom f = 4 and significance level α = 0.05;

^a^Mean ± SD (n = 6).

^b^Resolutions were calculated between two adjacent peaks.

^c^ normal distribution of residuals if p > 0.05.

Regression analysis results obtained for examined fluoroquinolones are presented in Table 1. The correlation coefficients (R) and determination coefficients (R^2^) obtained for linear model for all examined fluoroquinolones were greater than 0.999. The y-intercepts of the linear equation for CIP, MOX, NOR and OFL were statistically insignificant. The distribution of the residuals can well be approximated with a normal distribution as it is shown by p-values (p > 0.05) of the Shapiro-Wilk normality test. Based on regression analysis, it was assumed that the calibration data fitted well to linear model. Linearity range was observed in the wide concentration range 0.18–2.00 mg mL^-1^ for CIP, 0.18 – 1.50 mg mL^-1^ for MOX, 0.26 – 2.00 mg mL^-1^ for NOR and 0.30-1.50 mg mL^-1^ for OFL.

Sensitivity of the method was good. The LOD and LOQ values were found to be from 0.060 to 0.10 mg mL^-1^ and from 0.18 to 0.30 mg mL^-1^, respectively. Good precision and intermediate precision with %RSD less than 2.0% was observed. Detailed results were presented in Table 1. In all the deliberately varied chromatographic conditions (flow rate, column temperature), examined fluoroquinolones and degradation products were adequately resolved, and the order of elution remained unchanged.

### Photodegradation of examined fluoroquinolones in presence of excipients

The stability of the dosage form is different than API occurring separately, and therefore the stability studies for both forms are justified. In the case of tablets, pharmaceutical product contains additives that may affect the stability of the drug by speeding up or slowing down the degradation process.

In the available literature, only a few papers presenting the problem of photodegradation of the active ingredients in the presence of excipients in the solid phase are known [12]. Studies of the effect of UVA irradiation on the CIP, MOX, NOR and OFL in the solid phase in the presence of excipients from the tablets have shown that analyzed substances undergo photodegradation and differences occurring in the chromatograms concern only the number of peaks, peak area and RT values. However, no changes in the dark control samples were observed.

Photodegradation of CIP and OFL leads to formation of six photodegradation products CP-1 - CP-6 and OP-1 - OP-6, (Figure 1A and D), whereas in the case of NOR five photodegradation products were identified NP-1 - NP-5 (Figure 1C ). Peak areas of individual products increase with increasing exposure time. Photodegradation of MOX is significantly different compared with CIP, NOR and OFL because different number of decomposition products MP-1 and MP-10 (Figure 1B) appears during degradation process. Percentage amount of active substances in chromatograms also changes in a different way.

Photodegradation of CIP was 15.56%, OFL 11.91% and NOR 10.18% after 113 days of exposure, and for MOX 21.56% after 105 days of exposure.

Comparing the excipients present in the tested pharmaceutical preparation some certain similarities and differences regarding the inorganic components, which may be involved in the photodegradation process can be observed. The presence of TiO_2_ in the tablet mass in all tested pharmaceutical preparations and the presence of Fe_2_O_3_ in MOX pharmaceutical preparation is interesting from the photochemical point of view.

Titanium dioxide is one of the compounds having the ability to process the photon energy into chemical energy [19] and the Fe (III) ions, which may undergo an irreversible reduction during irradiation are considered as photosensitizers in photochemical reactions [20,21].

In the available literature manuscripts describing the increase in the efficiency of photo-degradation under the influence of the photocatalytic system TiO2/Fe (III) salt in comparison with the action of these compounds present separately are known [22,23].

The effect of Fe_2_O_3_ and TiO_2_ present in the MOX tablet mass on the acceleration of photodegradation compared to other fluoroquinolones in which there is only TiO_2_ can not therefore be excluded. The observed differences in photodegradation process may also be dependent on the chemical structure of individual fluoroquinolones.

### Kinetic evaluation

**Figure 2:**
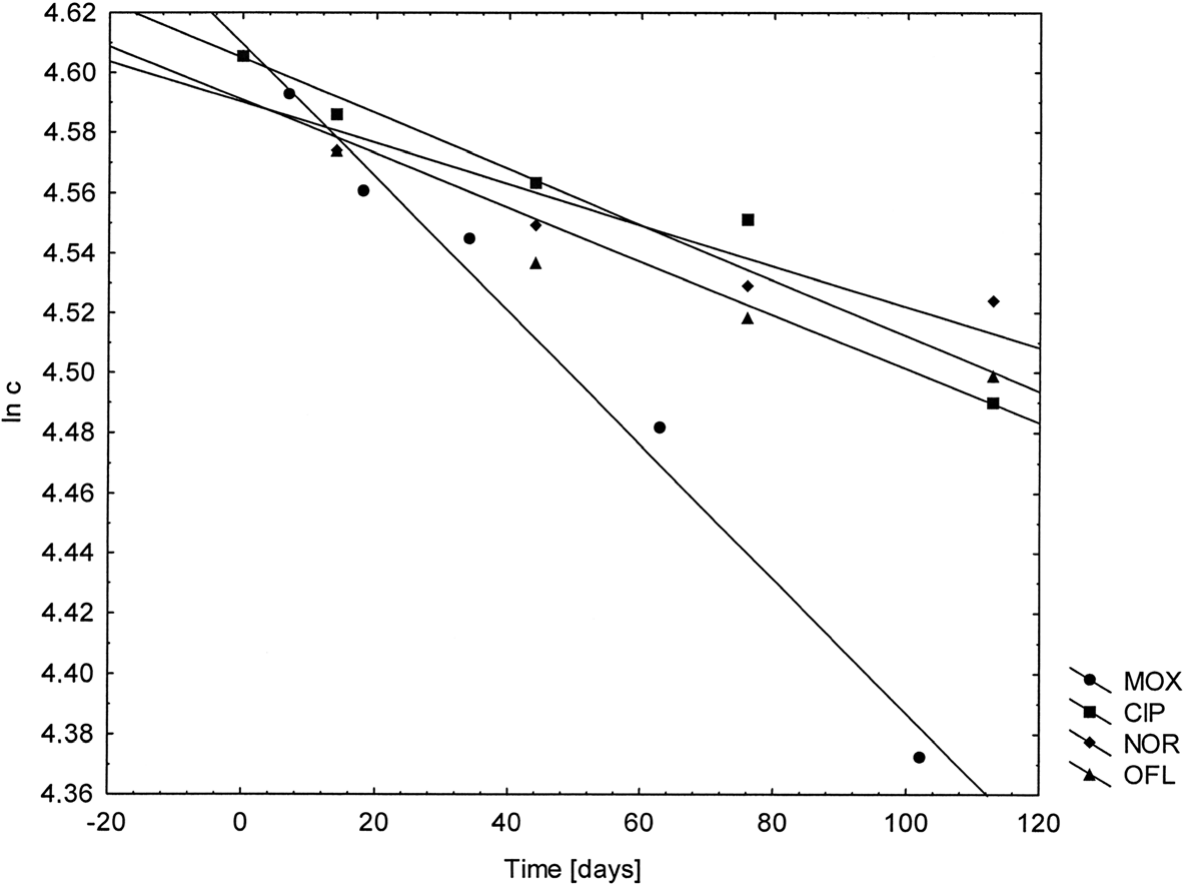
The ln c = f(t) graph of photodegradation of CIP, MOX, NOR and OFL.

The analysis of the equation ln c = f(t) for the photodegradation of CIP, MOX, NOR and OFL revealed that the process followed the kinetics of first order reaction (Figure 2).

Depending on the type of fluoroquinolone, distribution of the obtained results was observed (correlation coefficient R was from 0.9530 to 0.9936), which may be due to the presence of excipients that may reduce the intensity of radiation absorbed by the tested compounds.

The calculated reaction rate constants k take different values from the smallest for NOR, higher for CIP and OFL to the largest for MOX. Visible differences in photostability of tested compounds are also confirmed by t_0.1_ and t_0.5_ values (Table 2).

**Table 2:** The kinetic results of photodegradation

**Component**	**k [days**^**-1**^**]**	**t**_**0.1**_**[days]**	**t**_**0.5**_**[days]**	**Correlation coefficient r**
**CIP**	0.9× 10^-3^	117.00	770.00	0.9721
**MOX**	2.2× 10^-3^	47.86	315.00	0.9936
**NOR**	0.7× 10^-3^	150.43	990.00	0.9330
**OFL**	0.9×10^-3^	117.00	770.00	0.9619

Analyzing the photodegradation results for CIP, MOX, NOR and OFL in the powdered tablets it can be assumed that the tablets containing NOR will be the least susceptible to photodegradation process comparing to tablets with CIP and OFL and almost 3 times more photostable than tablets with MOX.

### Identification of photodegradation products

The identification of photoproducts of four fluoroquinolones (CIP, MOX, NOR, OFL) was performed on a basis of UPLC/MS analysis. Photodegradation products of fluoroquinolones are shown in Tables 3, 4, 5 and 6, for CIP, MOX, NOR, and OFL, respectively.

**Table 3:** Products of photodegradation of CIP

**Product Id**	**RT**	**[M + H]**^**+**^	**Proposed structure**
**CP**-**1**	2.12	330.1	
**CP**-**2**	2.25	378.1	
**CP**-**3**	2.37	348.1	
**CIP**	2.53	332.1	
**CP**-**4**	2.70	362.1	
**CP**-**5**	3.49	291.1	
**CP**-**6**	3.64	263.1	

**Table 4:** Products of photodegradation of MOX

**Product Id**	**RT**	**[M + H]**^**+**^	**Proposed structure**
**MP**-**1**	1.93	252.1	
**MP**-**2**	2.33	432.1	
**MP**-**3**	2.43	416.1	
**MP**-**4**	2.53	418.1	
**MP**-**5**	2.62	365.1	
**MP**-**6**	2.72	418.1	
**MP**-**7**	2.88	442.1	
**MP**-**8**	2.93	416.1	
**MP**-**9**	3.12	418.1	
**MOX**	3.26	402.1	
**MP**-**10**	4.10	293.1	

**Table 5:** Products of photodegradation of NOR

**Product Id**	**RT**	**[M + H]**^**+**^	**Proposed structure**
**NP**-**1**	2.28	294.1	
**NOR**	2.45	320.1	
**NP**-**2**	3.35	279.1	
**NP**-**3**	3.47	251.1	
**NP**-**4**	3.58	363.1	
**NP**-**5**	3.92	348.1	

**Table 6:** Products of photodegradation of OFL

**Product Id**	**RT**	**[M + H]**^**+**^	**Proposed structure**
**OFL**	2.50	362.1	
**OP**-**1**	2.81	378.1	
**OP**-**2**	3.07	307.1	
**OP**-**3**	3.38	392.1	
**OP**-**4**	3.64	279.1	
**OP**-**5**	3.97	376.1	
**OP-6**	5.32	309.1	

The photodegradation process was found mainly to affect 7-amine substituent, while the fluoroquinolone core remained unchanged. The only exception was MOX, which formed product of decarboxylation and partial reduction of dihydropiridine ring (MP-1).

Generally, the photodegradation proceeded with ring opening and dealkylation of 7-amine substituent of the investigated fluoroquinolones or hydroxylation in the close vicinity to nitrogen atoms in the substituent in the 7 position of the fluoroquinolones, successive oxidation to their oxo counterparts and subsequently opening the ring of the substituent.

The most abundant products were products of ring opening and dealkylation in the substituent in the 7 position, containing amine moiety (CP-6, MP-10, NP-3, OP-4) and their ethyl derivatives (CP-5, NP-2, OP-2). In case of NOR 2-aminoethyl derivative (NP-1) was also observed.

Less abundant products of the photodegradation of the fluoroquinolones were products of hydroxylation and subsequent oxidation in the close vicinity of nitrogen atoms of the substituent in the 7 position, containing one hydroxyl moiety (CP-3, MP-4, MP-6, MP-9, OP-1), their oxo counterparts (MP-3, MP-8 and OP-5), hydroxylactam moiety (CP-4, MP-2, OP-3) or multiple carbonyl groups (MP-7, NP-5).

Other routs of photodegradation led to degradation products with double bond on nitrogen in the substituent in the 7 position (CP-1), or containing partially degraded oxidized ring of that substituent (CP-2, MP-5, NP-4, OP-6).

### Differential Scanning Calorimetry

**Figure 3:**
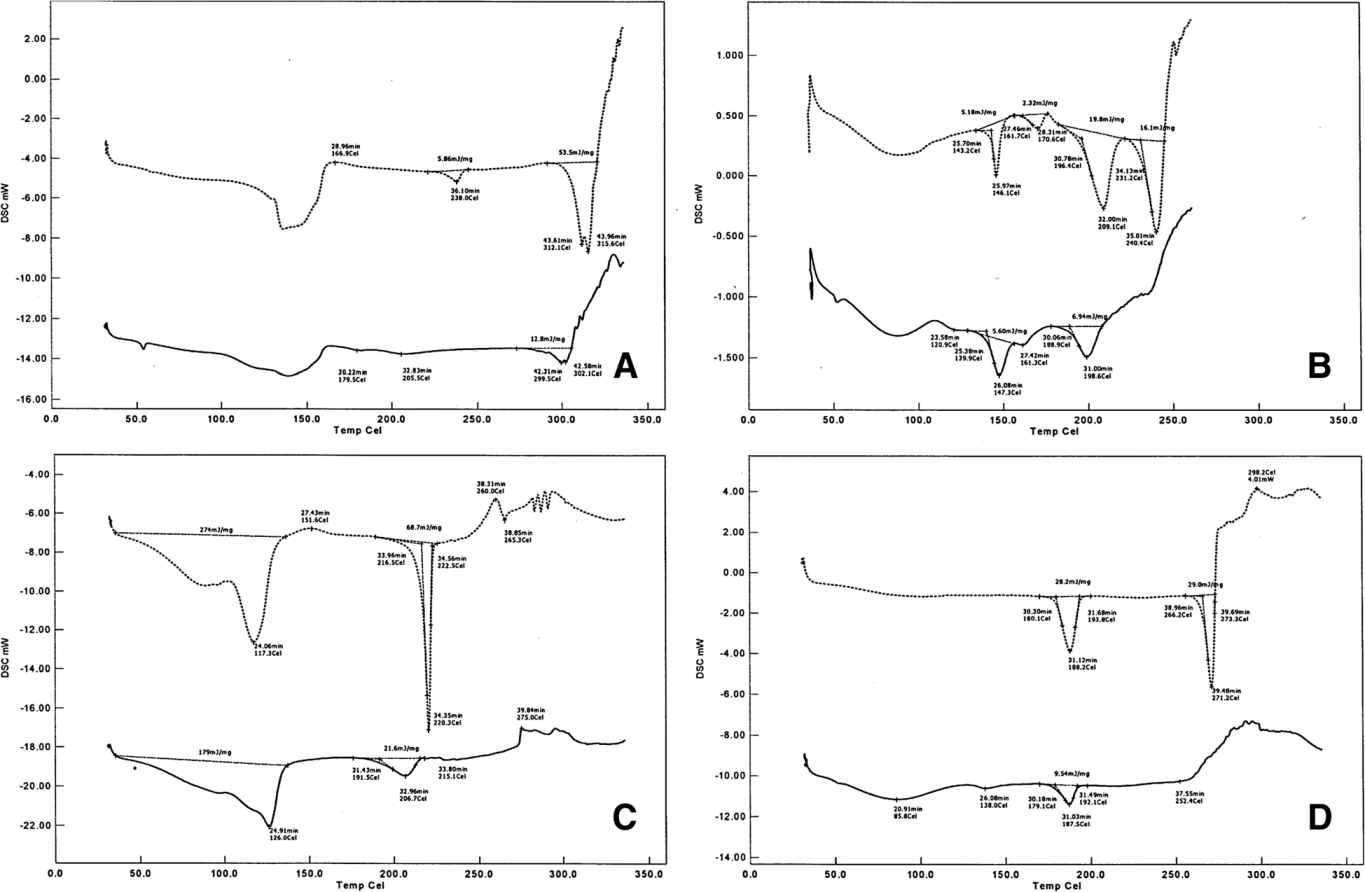
The DSC curves of the examined fluoroquinolones; dotted line: before exposure and continuous line: after exposure to UVA; A – CIP, B – MOX, C – NOR, D – OFL.

The sample of powdered CIP tablets exposed to UVA shows a number of similarities and differences in comparison to the sample not exposed (Figure 3A). In the temperature range of 104-174°C a broad endotherm, associated with the dehydration process, is observed [24] while there is no endothermic transition at T_max_ = 238.0°C [25]. Moreover, there are two small endothermic peaks at T_max_ = 179.5°C and T_max_ = 205.5°C coming from the melting processes of the newly created chemical compounds. Endothermic peak of simultaneous melting and decomposition process has a smaller value of enthalpy ΔH = 12.8 mJ mg^-1^ and has been moved toward lower temperatures of T_max_ = 299.5°C and T_max_ = 302.1°C.

The DSC curves of powdered NOR tablets, before and after UVA irradiation (Figure 3B), show a significant temperature T_onset_ and T_max_ shifts and a reductions of the corresponding values of enthalpy ΔH. The maximum temperature of endothermic peak corresponding to the maximum of the first stage of decomposition [26] was moved to 126.0°C and the enthalpy decreased to ΔH = 179.0 mJ mg^-1^. The next endothermic peak, describing the melting process T_onset_ = 220.3°C [27], was also changed. The value of enthalpy ΔH decreased to 21.6 mJ mg^-1^ and the melting temperatures T_onset_ and T_max_ are 191.5°C and 206.7°C respectively. The endo- and exothermic peaks located at the end of the DSC curves show different shapes. The exothermic peak presented in the not irradiated sample at T_max_ = 151.6°C was not found.

The DSC curve of MOX powdered tablets exposed to UVA shows the broadest changes compared to the corresponding not exposed sample (Figure 3C). One can see the absence of the last melting peak [28] at T_onset_ = 231.2°C and a rapid growth of a jagged baseline with tiny spikes. This observations suggest that the decomposition of the sample starts much earlier. Endothermic transitions at T_max_ 209.1°C and 170.6°C was moved toward lower values 198.6°C and 161.3°C. The enthalpies decreased as well. The peak at T_max_ = 146.1°C remains unchanged both for the temperature T_onset_ and ΔH values. Particularly noteworthy is a small endothermic process that occurs in the T_max_ = 120.9°C, which is probably related to the decomposition of the sample.

In contrast to the MOX, NOR and CIP the OFL compound seems to be more thermally stable and its decomposition, associated with weight loss, starts just after reaching the melting point T = 264.8°C [29]. The DSC curves obtained for powdered UVA irradiated and not irradiated tablets are not corresponding to each other (Figure 3D). While there is no endothermic melting peak at T_onset_ = 266.2°C, the new, small one at T_max_ = 138.0°C is observed. This peak could be considered as a degradation product. The peak at temperature of T_onset_ = 180.1°C and ΔH = 28.2 and mJ mg^-1^, under the influence of UVA light, significantly reduced the value of enthalpy to ΔH = 9.54 mJ mg^-1^ (Figure 3D).

Summing up these part of studies, one can see that DSC curves obtained for samples exposed to UVA light differ significantly from those not exposed. The broadest changes, comparing to the CIP, NOR and OFL, concerned the MOX, what confirms the results obtained by UPLC-MS/MS method.

## Conclusions

The developed UPLC-MS/MS method enables the determination of CIP, MOX, NOR and OFL in the presence of photodegradation products and identification of photodegradation products. The method meets the acceptance criteria for validation which guarantees correct analysis results. It has been shown that the tested fluoroquinolones occurring in the presence of excipients undergo photodegradation under the influence of UVA radiation. Photodegradation follows the kinetics of a first order reaction. The results obtained by UPLC-MS/MS and the calculated kinetic parameters k and t_0.1_ and t_0.5_ have shown that photodegradation of MOX is faster than CIP, NOR and OFL. Greater susceptibility of MOX to photodegradation process has also been shown by DSC method. It seems that the differences obtained during the photodegradation of tested fluoroquinolones may be connected with the presence of inorganic components in the tablet powder such as Fe_2_O_3_ and TiO_2_.

## Abbreviations

API: Active pharmaceutical ingredient; A: The slope of regression line; CAD: Collision activated dissociations; CIP: Ciprofloxacin; DSC: Differential scanning calorimetry; ESI: Electrospray ionization; ΔH: Enthalpy; K: Reaction rate constants; LOD: Limit of detection; LOQ: Limit of quantitation; MOX: Moxifloxacin; MS/MS: Tandem mass spectrometry; NOR: Norfloxacin; OFL: Ofloxacin; RT: Retention time; R: Correlation coefficient; R2: Determination coefficient; RSD: Relative standard deviation; Se: Standard error of residuals; UPLC: Ultra-performance liquid chromatography.

## Competing interests

The authors declare that they have no competing interests.

## Authors’ contributions

UH: Participated in the experimental designing, method validation, results discussion and writing of the manuscript. PŻ: Participated in experimental work and establishing the structure of the degradation products. PT: Performed experimental work by DSC method and contributed in results discussion. BŻ-W: contributed in reviewing the literature, and experimental work. JK: contributed in results discussion and revised the manuscript. All authors have read and approved the final manuscript.
